# Diagnostic performance of SARC-F and SARC-CalF in screening for sarcopenia in older adults in Northern Brazil

**DOI:** 10.1038/s41598-023-39002-y

**Published:** 2023-07-20

**Authors:** Alex Barreto de Lima, Gustavo dos Santos Ribeiro, Duarte Henriques-Neto, Élvio Rúbio Gouveia, Fátima Baptista

**Affiliations:** 1 CIPER, Faculdade de Motricidade Humana, Universidade de Lisboa, Lisbon, Portugal; 2 Universidade do Estado do Amazonas, Amazonas, Brazil; 3 Programa de Pós-Graduação em Ciências da Reabilitação, Universidade Federal de Ciências da Saúde de Porto Alegre, Porto Alegre, Brazil; 4 Research Center in Sports Sciences, Health Sciences and Human Development, University of Maia, Maia, Portugal; 5 Department of Physical Education and Sport, University of Madeira, Funchal, Portugal; 6 LARSYS, Interactive Technologies Institute, Funchal, Portugal

**Keywords:** Geriatrics, Outcomes research

## Abstract

To compare the performance of SARC-F and SARC-CalF as screening tools for sarcopenia. Cross-sectional study with a convenience sample of 312 community-dwelling older people. Sarcopenia was defined as low handgrip strength (HGS) or low gait speed (GS ≤ 0.8 m/s). HGS was measured by dynamometry and GS by the 4-m walking speed test. For HGS, six criteria (C) were used to identify sarcopenia in men/women: C_I_: < 27 kg/16 kg; C_II_: < 35.5 kg/20.0 kg; C_III_: grip over body mass index < 1.05/< 0.79; C_IV_: grip strength over total body fat < 1.66/< 0.65; C_V_: grip over bodyweight < 0.45/< 0.34; C_VI_: < 27 kg/16 kg and low skeletal muscle mass index (SMMI); C_I_ and C_VI_ defined according to the European Working Group on sarcopenia in older people and the rest according to the sarcopenia definition and outcomes Consortium. For sarcopenia screening, the SARC-F (≥ 4 points) and the SARC-CalF (≥ 11 points) were used. The kappa analysis revealed no agreement between the SARC-F and the various criteria for the identification of sarcopenia in men. The same lack of agreement was observed in women with some exceptions: C_I_ = 0.161 ± 0.074, *p* = 0.020; GS = 0.209 ± 0.076, *p* = 0.003. Concerning the Cohen’s kappa between the SARC-Calf and the reference criteria of sarcopenia, the following coefficients were observed as significant for women: C_I_ = 0.201 ± 0.069, *p* = 0.003; C_II_ = 0.186 ± 0.064, *p* = 0.005; GS = 0.273 ± 0.068, *p* = 0.0001; and for men: C_II_ = 0.139 ± 0.053, *p* = 0.021; GS = 0.223 ± 0.099, *p* = 0.011. ROC curves revealed the SARC-Calf with acceptable discrimination and reasonable sarcopenia predictive capacity considering a cutoff value of 10.5 in both men (AUC: 67.5%, *p* = 0.022; Se = 52.9%; Sp = 76.8%) and women (AUC: 72.4%, *p* < 0.001; Se = 63%; Sp = 68.5%) concerning GS. The SARC-CalF performed better than the SARC-F for screening sarcopenia in the population ≥ 60 years of age in the Amazonas, measured through walking slowness.

## Introduction

The life expectancy of the human population has been increasing worldwide, which is to be welcomed. However, this increase may not correspond to a more significant number of years of healthy life as aging is the expression of a continuous biological process associated with a decrease in the function of different bodily systems^1–3^. The loss of skeletal muscle mass and muscle function (strength and performance) with aging or from other secondary causes characterizes sarcopenia, a disease established by the International Classification of Diseases-10 code in 2016^4,5^. Sarcopenia is associated with an increased risk of falls, fractures, physical disability, higher morbidity, and death^5,6^. These associations reveal the need for help and treatment services for older adults with sarcopenia in a community, institutional, or hospital context and the associated costs^7^. Sarcopenia can be prevented and reversed^8,9^. However, simple approaches capable of discriminating against suspected cases of sarcopenia are needed^10^.

In 2010 the European Working Group on Sarcopenia in Older People (EWGSOP) proposed an approach to diagnosing sarcopenia that combined low muscle mass with low strength or low physical performance^7^. The diagnosis was recently updated by the same group (EWGSOP2), evolving into an evaluation of sarcopenia in three steps: identification (low strength), confirmation (low muscle mass), and degree of severity (low physical performance), preceded by a screening questionnaire^11,12^. In 2020, another working group, the Sarcopenia Definition and Outcomes Consortium (SDOC), emphasizes muscle strength and physical performance for the diagnosis of sarcopenia but does not recommend the assessment of muscle mass for the diagnosis of sarcopenia because muscle mass (assessed by dual-energy x-ray absorptiometry or by bioimpedance) does not seem to be a predictor of the risk of functional disability^13,14^. Screening for sarcopenia, based on symptomatology and the occurrence of events is, however, the first step in assessing sarcopenia suggested by both approaches^15,16^. This process precedes the objective assessment in case of suspicion. To this end, the SARC-F questionnaire is the most popular.

The SARC-F questionnaire has been validated for different languages^9,10,15,17–19^ and clinical settings^9,15,17,20,21^ using several gold standard diagnostic modalities as a reference^22,23^. SARC-F is a simple, easy-to-use, 5-item sarcopenia screening questionnaire^8^, where five domains are included in the questionnaire: (1) Strength, (2) Assistance with walking, (3) Rising from a chair, (4) Climbing stairs, and (5) Falls^24^. Since 2018, SARC-F has been part of the sarcopenia diagnostic algorithm proposed by the European Working Group on Sarcopenia in Older Adults 2 (EWGSOP2)^25^.

However, the performance of the SARC-F for screening sarcopenia has been shown to be highly variable^26^, with the sensitivity for suspected cases of sarcopenia ranging from 3.9 to 95.4%^27^. The high variability, with poor to fair diagnostic accuracy, and greater specificity than sensitivity^19,22–24,26,28^ constitutes a limitation to correctly identifying positive cases of sarcopenia^29^. It is postulated that the low sensitivity of SARC-F for the suspicion of sarcopenia is because it does not require information on any muscle mass marker^13,30,31^. In light of this assumption, two modified versions of the SARC-F emerged with the inclusion of other markers, a more specific marker such as CalF circumference (SARC-CalF), but also less specific markers such as age and body mass index (SARC-F + EBM)^24^.

To identify sarcopenia symptoms the SARC-F, and SARC-CALF are instruments widely validated worldwide^32^. However, when compared with each other, the SARC-CalF demonstrated greater sensitivity (66.7% vs. 33.3%), high discrimination (AUC: 0.736 vs. AUC: 0.592), and a similar specificity (82.9% vs. 84.2%)^24^. Based on data the SARC-F can better classify non-sarcopenic than sarcopenic older adult populations^33^. Additionally, CalF circumference measurements (SARC-CalF) showed that this specific variable improves the screening for sarcopenia^16,29,34,35^. by increasing its sensitivity relative to SARC-F^36,37^.

However, it is necessary to analyze the performance of sarcopenia screening instruments regarding the various objective criteria for assessing muscle function (strength and performance) proposed by the different working groups. Thus, the present study aimed to compare the performance of SARC-F and SARC-CalF as approaches for the screening of sarcopenia using the criteria proposed by the EWGSOP2 and the SDOC as a reference for the diagnosis of sarcopenia in older adults from the state of Amazonas, Brazil^13^.

## Methods

### Design and study population

**Figure 1:**
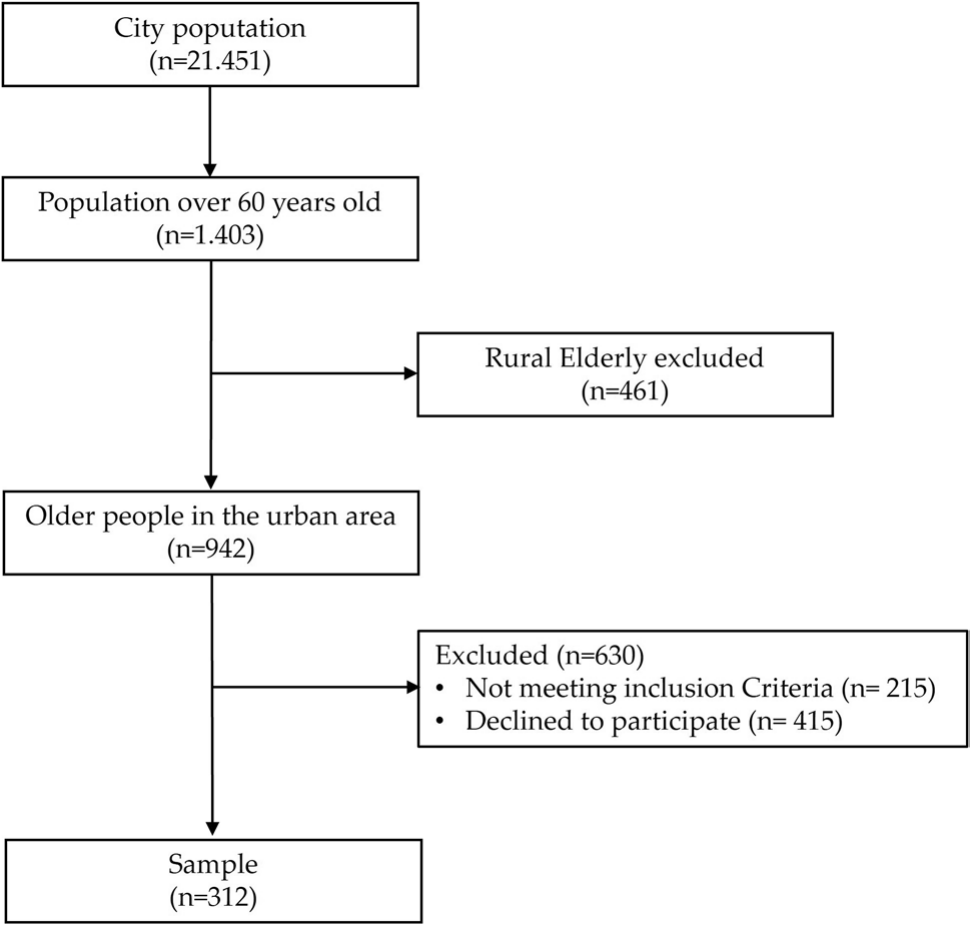
Flow chart of the study.

This work is based on a cross-sectional study with a convenience sample involving 312 community-dwelling older adults (64% women) living in the urban area of the city of Novo Aripuanã (Amazonas), Northern Brazil. Older adults living in rural areas were excluded from the research due to difficulty accessing the evaluation site (distance and necessary means of transport) (Fig. 1). The sample size was calculated using GPower (Heinrich-Heine-University, Düsseldorf, Germany; software 3.1.9.7)^38^. Calculations were based on direct logistic regression, family of z tests, considering an odds ratio of 1.5 and α = 0.05, and a computational power of 0.95. The following criteria were considered for the inclusion of participants: (a) older adults aged 60 or over living in the urban area of the city; (b) be independent in carrying out activities of daily living; (c) moderate or high level of cognitive functioning; (d) without chest pain and/or angina pectoris and limiting joint pain^39^. The Mini-Mental State Examination (MMSE) evaluated the cognitive criteria selection^40^. An MMSE ≤ 15/30 points were used to exclude the participants of study^40^. Before the data collection process, all procedures and potential risks were explained and informed consent forms were signed by all participants. This study was conducted in accordance with the Declaration of Helsinki^41^. After selecting the participants, the following evaluations were performed: sociodemographic, anthropometric, muscle function (strength and performance) and sarcopenia symptoms.

This study was approved by the Ethics Committee of the State UEA and the study was carried out in accordance with the Declaration of Helsinki and Resolution 466/12 of the National Health Council, making part of the research project: “Sarcopenic Syndrome-Physical Function, Phenotype and Quality of Life” (CAAE 74055517.9.0000.5016/Referee 2.281.400).

### Sociodemography

The questionnaire from the Brazilian Association of Research Companies was used to assess the sociodemographic variables^42^. The questionnaire classifies individuals into five social classes, ranging from class A (those with higher purchasing power) to class E (those with lower purchasing power) based on ownership of some consumer goods, head's schooling family, and access to public services.

### Anthropometric and body composition characteristics

Body height and body mass were determined using a mechanical scale with the stadiometer attached, with the older adults barefoot and wearing light clothing. The categorization of body mass index (BMI) followed the guidelines of the World Health Organization^28^. The CalF circumference is a marker of muscle mass and was measured with an inelastic metallic tape measure, the measurement was taken at the point of the greatest circumference with the individual seated, with the leg forming a 90° angle and feet flat on the ground^30^ without compressing subcutaneous tissues and was used to calculate the SARC-CalF. Skeletal muscle mass (SMM) was estimated according to Lee and colleagues^43^ using the corrected arm, thigh, and CalF circumferences and normalized for body height (SMMI, kg/m^2^). To identify participants with low SMMI, the cutoff values proposed by Walowski and colleagues were used^44^. The cutoff values align with those recommended by the EWGSOP (-2 SD below the healthy young adult population) but are adjusted for the BMI.

### Sarcopenia identification-muscle function (strength and performance)

For the identification of sarcopenia, muscle function, namely handgrip strength and performance (gait speed), were considered according to the criteria of the EWGSOP2 and SDOC^13,25^. Handgrip strength was measured using a digital hand dynamometer (Camry EH10; Sensun Weighing Apparatus Group Ltd., Guangdong, China)^45^ following the procedures recommended by Roberts and colleagues^31^. The assessment was performed sitting with the elbow flexed at 90 degrees. Both the left and right arms were measured twice. Two measurements were performed for each hand alternately, and the highest value found among the four measures was used. The results were recorded in kilograms (kg). For the diagnosis of sarcopenia through the handgrip strength, six criteria were considered, the first and the sixth criteria according to the EWGSOP2^25^ (I) < 27 kg in men and < 16 kg in women, (VI) < 27 kg in men and < 16 kg in women and low SMMI; and the remaining criteria according to SDOC^13^: (II) < 35.5 kg in men and < 20.0 kg in women; (III) grip over body mass index < 1.05 for men and 0.79 for women; (IV) grip strength over total body fat < 1.66 for men and < 0.65 for women; (V) grip over body weight < 0.45 for men and < 0.34 for women. The usual walking speed (criterion VII) was evaluated at a 4 m distance^15^. The test was performed twice, adopting the best execution time. Values below 0.8 m/s, regardless of sex, were considered indicative of decreased physical performance because it is the most consensual cutoff value by the various sarcopenia working groups, except for the International Working Group on Sarcopenia (IWGS: < 1.0 m/s)^13,15,46,47^.

### Sarcopenia suspicion-symptoms

The signalling of possible cases of sarcopenia was performed through SARC-F and SARC-CalF. The SARC-F is a 5-item questionnaire that asks about difficulties in strength, walking, getting up from a chair, climbing stairs, and history of falls. A score ≥ 4 points on the SARC-F is suggestive of sarcopenia^48^. For this purpose, the translated and validated version of the SARC-F for the Brazilian population was used^29^. The SARC-CalF consists of the SARC-F complemented with a measurement of CalF circumference. The SARC-CalF score ranges from 0 to 20 points, with a score ≥ 11 points suggestive of sarcopenia^29^: men and women with CalF circumference < 34 cm and < 33 cm, respectively (suggestive of low muscle mass) receive a 10-point increase from the original SARC-F score.

### Statistical analysis

All statistical analyses were performed using SPSS version 26.0 for Windows software (SPSS, Chicago, IL, USA). Data were stratified by sex and described as the absolute and relative frequency of cases (n, %) and mean + standard deviation (SD). The Chi-Square Test, the Mann–Whitney test, and Fisher’s exact test were used to compare continuous and nominal characteristics of the sample between the sexes, respectively. Data normality was verified with the Kolmogorov–Smirnov. The accuracy of the screening tools (SARC-F and SARC-CalF) was evaluated through K analysis and the area under the curve (AUC), sensitivity (Se), specificity (Sp), positive predictive value (PPV), and negative predictive value (NPV) analysis. The significance level was set at *p* ≤ 0.05.

## Results

The sociodemographic and anthropometric characteristics, the sarcopenia symptoms, and markers of sarcopenia (muscle strength and performance) of the sample are described in Table 1. Our sample was predominantly female, aged > 70 yrs, non-literate, and of socioeconomic class D/E. Most participants were overweight (42.0%) and obese (21.2%). No sex differences were observed for sarcopenia symptoms, assessed using the SARC-F and SARC-CalF, despite differences in CalF circumference, muscle strength, and performance, with men showing better results than women in these variables.

**Table 1:** Characteristics of the participants by sex: sociodemographic, body composition, sarcopenia symptoms, and sarcopenia biomarkers (muscle function: strength and gait speed).

Variables	Overall	Men	Women	*p*-value
Sample size, n (%)	312	112 (35.9)	200 (64.1)	
Age, years				0.122*
60–69	132 (42.3)	39 (34.8)	93 (46.5)	
70–79	120 (38.5)	50 (44.6)	70 (35.0)	
> 80	60 (19.2)	23 (20.5)	37 (18.5)	
Educational level				0.123*
Non-literate, n (%)	176 (56.4)	63 (56.3)	113 (56.5)	
Elementary school, n (%)	83 (26.6)	29 (25.9)	54 (27.0)	
High school, n (%)	28 (9.0)	6 (5.4)	22 (11.0)	
Graduate or above, n (%)	25 (8.0)	14 (12.5)	11 (5.5)	
Socioeconomic status class, n (%)				0.615^†^
C	18 (5.8)	5 (4.5)	13 (6.5)	
D/E	294 (94.2)	107 (95.5)	187 (93.5)	
Body composition				
Body mass, kg	63.7 ± 12.7	69.3 ± 11.6	60.5 ± 12.2	< 0.001**
Body height, cm	153.7 ± 8.2	160.0 ± 8.3	150.1 ± 5.7	< 0.001**
BMI, kg/m^2^ (%)				0.131*
Underweight	6 (1.9)	0 (0)	6 (3.0)	
Normal weight	109 (34.9)	37 (33.0)	72 (36.0)	
Overweight	131 (42.0)	54 (48.2)	77 (38.5)	
Obese	66 (21.2)	21 (18.8)	45 (22.5)	
SMMI (kg/m^2^)	8.34 ± 1.47	9.23 ± 1.16	7.84 ± 1.39	< 0.001**
Sarcopenia symptoms				
SARC-F (pts)	1.75 ± 1.88	1.43 ± 1.68	1.92 ± 1.95	0.025**
SARC-CalF (pts)	7.2 ± 5.5	6.42 ± 5.12	7.57 ± 5.71	0.080**
Calf circunference, cm	32.8 ± 3.4	33.8 ± 3.0	32.2 ± 3.5	< 0.001**
Muscle function				
Handgrip strength, kg	23.7 ± 9.2	31.4 ± 8.9	19.3 ± 5.9	< 0.001**
Gait speed, m/s	1.1 ± 0.4	1.2 ± 0.4	1.0 ± 0.4	< 0.001**

BMI, body mass index; SMMI, skeletal muscle mass index; SARC-F, sarcopenia screening questionnaire; SARC-Calf, sarcopenia screening questionnaire adding calf circumference.

**Test t student independent; *Chi-Square test; ^†^Fisher's exact test.

The prevalence of sarcopenia assessed through symptoms and muscle function are described in Tables 2 and 3. Sarcopenia was suspected in 12.5% of men and 21% of women when evaluated by the SARC-F (Table 2) and in 27.7% of men and e 40% of women when evaluated by SARC-CalF (Table 3). Regarding the cross-classification analysis between sarcopenia symptoms and muscle function, the kappa analysis revealed no agreement between the SARC-F and the various criteria of handgrip strength (C_I–VI_) and gait speed (C_VII_) for the identification of sarcopenia in men (Table 2): K_I_ = 0.139 ± 0.090, *p* = 0.087; K_II_ = 0.039 ± 0.034, *p* = 0.322; K_III_ = − 0.021 ± 0.072, *p* = 0.770; K_IV_ = − 0.049 ± 0.082, *p* = 0.590; K_V_ = 0.036 ± 0.062, *p* = 0.568; K_VI_ = − 0.014 ± 0.029, *p* = 0.548; K_VII_ = 0.065 ± 0.105, *p* = 0.486. The same lack of agreement was observed in women except for handgrip strength C_I_ and gait speed C_VII_: K_I_ = 0.161 ± 0.074, *p* = 0.020; K_II_ = 0.067 ± 0.051, *p* = 0.200; K_III_ = 0.038 ± 0.045, *p* = 0.403; K_IV_ = − 0.101 ± 0.063, *p* = 0.146; K_V_ = 0.079 ± 0.052, *p* = 0.135; K_VI_ = − 0.004 ± 0.030, *p* = 0.892; K_VII_ = 0.209 ± 0.076, *p* = 0.003.

**Table 2:** Cross classification analysis between suspected cases of sarcopenia through SARC-F and muscle function by sex.

Criteria	SARC-F					
	Men			Women		
	− cases	+ cases	Total	− cases	+ cases	Total
C_I__LowHandgrip_EWGSOP2_						
− cases	63.4%	6.3%	69.6%	59.5%	12.0%	71.5%
+ cases	24.1%	6.3%	30.4%	19.5%	9.0%	28.5%
C_II__LowHandgrip_SDOC_						
− cases	23.2%	1.8%	25.0%	35.0%	7.0%	42.0%
+ cases	64.3%	10.7%	75.0%	44.0%	14.0%	58.0%
C_III__LowHandgrip/BMI						
− cases	52.7%	8.0%	60.7%	28.0%	6.0%	34.0%
+ cases	34.8%	4.5%	39.3%	51.0%	15.0%	66.0%
C_IV__LowHandgrip/FM						
− cases	69.6%	10.7%	80.4%	55.0%	17.0%	72.0%
+ cases	17.9%	1.8%	19.6%	24.0%	4.0%	28.0%
C_V__LowHandgrip/BM						
− cases	44.6%	5.4%	50.0%	36.5%	7.0%	43.5%
+ cases	42.9%	7.1%	50.0%	42.5%	14.0%	56.5%
C_VI__LowHandgrip_EWGSOP2_ and LowSMMI						
− cases	6.3%	2.7%	8.9%	11.0%	4.0%	15.0%
+ cases	33.9%	2.7%	36.6%	18.0%	3.0%	21.0%
C_VII__LowGaitSpeed						
− cases	75.0%	9.8%	84.8%	61.5%	11.5%	73.0%
+ cases	12.5%	2.7%	15.2%	17.5%	9.5%	27.0%

C, criterion; SARC-F, sarcopenia screening questionnaire; BM, body mass; BMI, body mass index; FM, fat mass; SMMI, skeletal muscle mass index; EWGSOP, European Working Group on Sarcopenia in Older People; SDOC, Sarcopenia Definition and Outcomes Consortium.

**Table 3:** Cross classification analysis between suspected cases of sarcopenia through SARC-CalF and muscle function by sex.

Criteria	SARC-Calf					
	Men			Women		
	− cases	+ cases	Total	− cases	+ cases	Total
C_I__LowHandgrip_EWGSOP2_						
− cases	53.6%	16.1%	69.6%	47.5%	24.0%	71.5%
+ cases	18.8%	11.6%	30.4%	12.5%	16.0%	28.5%
C_II__LowHandgrip_SDOC_						
− cases	22.3%	2.7%	25.0%	30.0%	12.0%	42.0%
+ cases	50.0%	25.0%	75.0%	30.0%	28.0%	58.0%
C_III__LowHandgrip/BMI						
− cases	44.6%	16.1%	60.7%	19.5%	14.5%	34.0%
+ cases	27.7%	11.6%	39.3%	40.5%	25.5%	66.0%
C_IV__LowHandgrip/FM						
− cases	57.1%	23.2%	80.4%	42.5%	29.5%	72.0%
+ cases	15.2%	4.5%	19.6%	17.5%	10.5%	28.0%
C_V__LowHandgrip/BM						
− cases	36.6%	13.4%	50.0%	23.5%	20.0%	43.5%
+ cases	35.7%	14.3%	50.0%	36.5%	20.0%	56.5%
C_VI__LowHandgrip_EWGSOP2_ and LowSMMI						
− cases	5.4%	3.6%	8.9%	7.5%	7.5%	15.0%
+ cases	25.0%	11.6%	36.6%	15.0%	6.0%	21.0%
C_VII__LowGaitSpeed						
− cases	65.2%	19.6%	84.8%	50.0%	23.0%	73.0%
+ cases	7.1%	8.0%	15.2%	10.0%	17.0%	27.0%

C, criterion; SARC-Calf, sarcopenia screening questionnaire adding calf circumference; BM, body mass; BMI, body mass index; FM, Fat Mass; SMMI, skeletal muscle mass index; EWGSOP2, European Working Group on Sarcopenia in Older People; SDOC, Sarcopenia Definition and Outcomes Consortium.

Concerning the kappa analysis between the SARC-CalF and the different criteria of handgrip strength and gait speed for the identification of sarcopenia, there were more agreements (Table 3). In women, agreement of SARC-CalF with handgrip strength criteria C_I_ and C_II_ and with the gait speed criterion C_VII_ was observed with the following K coefficients: K_I_ = 0.201 ± 0.069, *p* = 0.003; K_II_ = 0.186 ± 0.064, *p* = 0.005; K_III_ = − 0.034 ± 0.062, *p* = 0.583; K_IV_ = − 0.031 ± 0.068, *p* = 0.653; K_V_ = − 0.101 ± 0.067, *p* = 0.130; K_VI_ = − 0.009 ± 0.040, *p* = 0.815, K_VII_ = 0.273 ± 0.068, *p* = 0.0001. In men, it was found concordance of SARC-CalF with the handgrip criterion CII and with the gait speed criterion C_VII_: K_I_ = 0.155 ± 0.098, *p* = 0.099; K_II_ = 0.139 ± 0.053, *p* = 0.021; K_III_ = − 0.032 ± 0.092, *p* = 0.722; K_IV_ = − 0.053 ± 0.088, *p* = 0.563; K_V_ = 0.018 ± 0.085, *p* = 0.833; K_VI_ = − 0.047 ± 0.37, *p* = 0.183; K_VII_ = 0.223 ± 0.099, *p* = 0.011. Since K values below 0.2 are considered poor, we highlight the agreement between SARC-CalF and gait speed criterion C_VI_ in both sexes.

AUC, sensitivity, specificity, and positive and negative predictive values of SARC-F and SARC-CalF for sarcopenia screening according to different criteria of muscle function (handgrip strength and gait speed), are presented in Tables 4 and 5, respectively. The SARC-F did not reveal any ability to discriminate sarcopenia in men, regardless of the muscle function variable (muscle strength or gait speed) taken as a reference. In women, the SARC-F showed a poor discrimination ability with AUC values of 0.665 for criterion C_I_, 0.651 for C_II_, and 0.660 for C_VII_. When SARC-CalF was used to screen for sarcopenia in women, the corresponding AUC for the same reference criteria were 0.631 for C_I_, 0.641 for C_II_, and 0.724 for C_VII_. The SARC-CalF, in turn, was able to discriminate sarcopenia in men using the criteria C_II_ (AUC: 0.676) and C_VII_ (AUC: 0.675) as references. Generally, an AUC > 0.9 indicates exceptional discrimination, 0.7‒0.9 indicates moderate discrimination, 0.5‒0.7 indicates poor discrimination, and < 0.5 indicates result at chance^49^. In the significant models, the predictive power was reasonable, with the specificity consistently higher than the sensitivity and consequently higher values of NPV than PPV. Greater sensitivity for suspected sarcopenia was observed with the SARC-CalF in women with gait speed criterion C_VII_ (Se = 63.0) and men with handgrip criterion C_II_ (Se = 60.7) as references; the cutoff for these criteria was 10.5 pts and 4.5 pts, respectively.

**Table 4:** Sensitivity, specificity, positive and negative predictive values of SARC-F for screening sarcopenia according to different criteria.

Criteria	Cutoff	Sensitivity	Specificity	PPV	NPV	AUC	*p*
Men							
C_I__LowHandgrip_EWGSOP2_	–	–	–	–	–	0.535	0.552
C_II__LowHandgrip_SDOC_	–	–	–	–	–	0.599	0.119
C_III__LowHandgrip/BMI	–	–	–	–	–	0.488	0.828
C_IV__LowHandgrip/FM	–	–	–	–	–	0.506	0.933
C_V__LowHandgrip/BM	–	–	–	–	–	0.537	0.503
C_VI__LowHandgrip_EWGSOP2_ and LowSMMI	–	–	–	–	–	0.624	0.134
C_VII__LowGaitSpeed	–	–	–	–	–	0.589	0.241
Women							
C_I__LowHandgrip_EWGSOP2_	2.5	47.4 (34.0–61.0)	73.4 (65.4–80.5)	31.0 (22.5–41.1)	83.1 (78.8–86.6)	0.665	< 0.001
C_II__LowHandgrip_SDOC_	1.5	57.0 (47.4–66.1)	68.0 (56.8–77.6)	24.1 (19.8–29.1)	83.3 (75.9–88.8)	0.651	< 0.001
C_III__LowHandgrip/BMI	1.5	52.3 (43.4–61.0)	64.7 (52.2–75.9)	22.7 (19.0–26.9)	82.8 (73.5–88.7)	0.618	0.006
C_IV__LowHandgrip/FM	–	–	–	–	–	0.435	0.155
C_V__LowHandgrip/BM	1.5	54.0 (44.4–63.4)	63.2 (52.2–73.3)	24.8 (20.3–29.9)	83.9 (76.7–89.2)	0.612	0.006
C_VI__ Handgrip_EWGSOP2_ and LowSMMI	–	–	–	–	–	0.556	0.262
C_VII__LowGaitSpeed	2.5	51.9 (37.8–65.7)	74.7 (66.8–81.5)	35.2 (25.8–45.8)	84.2 (80.1–87.7)	0.660	< 0.001

C, criterion; EWGSOP2, European Working Group on Sarcopenia in Older People; SDOC, Sarcopenia Definition and Outcomes Consortium; CIII, grip strength over body mass index; CIV, grip strength over total body fat; CV, grip strength over body mass; SMMI, skeletal muscle mass index; PPV, predictive positive values; NPV, negative predictive values.

**Table 5:** Sensitivity, specificity, positive and negative predictive values of SARC-CalF for screening sarcopenia according to different criteria.

Criteria	Cutoff	Sensitivity	Specificity	PPV	NPV	AUC	*p*
Men							
C_I__LowHandgrip_EWGSOP2_	–	–	–	–	–	0.606	0.076
C_II__LowHandgrip_SDOC_	4.5	60.7 (49.5–71.2)	60.7 (40.6–78.5)	33.3 (29.3–37.6)	89.3 (73.0–96.2)	0.676	0.005
C_III__LowHandgrip/BMI	–	–	–	–	–	0.472	0.621
C_IV__LowHandgrip/FM	–	–	–	–	–	0.501	0.991
C_V__LowHandgrip/BM	–	–	–	–	–	0.441	0.280
C_VI__Low Handgrip_EWGSOP2_ and LowSMMI	–	–	–	–	–	0.484	0.792
C_VII__LowGaitSpeed	10.5	52.9 (27.8–77.0)	76.8 (67.1–84.9)	52.9 (32.3–72.6)	76.8 (72.4–80.8)	0.675	0.022
Women							
C_I__LowHandgrip_EWGSOP2_	10.5	56.1 (42.4–69.3)	66.4 (58.1–74.1)	56.9 (46.1–67.1)	66.9 (62.2–71.3)	0.631	0.004
C_II__LowHandgrip_SDOC_	10.5	48.3 (38.9–57.7)	71.4 (60.5–80.8)	48.3 (42.6–54.0)	71.4 (63.1–78.5)	0.641	0.001
C_III__LowHandgrip/BMI	–	–	–	–	–	0.456	0.310
C_IV__LowHandgrip/FM	–	–	–	–	–	0.485	0.740
C_V__LowHandgrip/BM	–	–	–	–	–	0.432	0.100
C_VI__Low Handgrip_EWGSOP2_ and LowSMMI	–	–	–	–	–	0.567	0.108
C_VII__LowGaitSpeed	10.5	63.0 (48.7–75.7)	68.5 (60.3–75.9)	63.0 (51.4–73.2)	68.5 (63.9–72.7)	0.724	0.001

C, criterion; EWGSOP2, European Working Group on Sarcopenia in Older People; SDOC, Sarcopenia Definition and Outcomes Consortium; CIII, grip strength over body mass index; CIV, grip strength over total body fat; CV, grip strength over body mass; SMMI, skeletal muscle mass index; PPV, predictive positive values; NPV, negative predictive values.

## Discussion

This study aimed to compare SARC-F and SARC-CalF tests as approaches for screening sarcopenia in older people. This is the first study examining these questionnaires in populations with specific characteristics, such as older adults from Amazonas. These are inexpensive, easy to administer, and minimally invasive approaches that assess the symptoms of sarcopenia (SARC-F) complemented by the CalF circumference as a possible indicator of muscle mass (SARC-CalF)^10^. This type of analysis is essential because there still needs to be a consensus on evaluating sarcopenia, including screening, among the different working groups dedicated to this subject^7,50–54^.

A greater suspicion of sarcopenia was observed when screening was performed with SARC-CalF: sarcopenia was detected by SARC-CalF in 27.7% of men and 40.0% of women, and SARC-F in 12.5% of men and 21.0% of women. Taking handgrip strength and gait speed as references, the prevalence of sarcopenia ranged between 15.2 and 75.0% for men and 27% and 66% for women, with the lowest prevalence associated with gait speed and the highest prevalence with handgrip in both sexes. From the analyzes carried out on the agreement between the measures for the suspicion (symptoms) and the identification (muscle function) of sarcopenia, a poor to fair diagnostic accuracy was observed in both sexes when the screening instrument was the SARC-CalF (AUC: 0.631–0.724, *p* < 0.01).

The SARC-F did not show any ability to discriminate against sarcopenia in men. The predictive ability of SARC-CalF was reasonable with sensitivity and specificity values above 50%, considering a cutoff value of 10.5 in both men and women when gait speed C_VII_ was the reference criteria for muscle function. As in most studies on the ability to predict sarcopenia from SARC-F or SARC-CalF, specificity was superior to sensitivity, meaning there were few false negative results than false positive results^9,15,18,24^. Low sensitivity implies that many subjects with sarcopenia will not be detected if assessed using these questionnaires. Specificity relates to the test’s ability to reject subjects without a condition correctly. Therefore, if not detected from SARC-F or SARC-CalF, sarcopenia can be ruled out without further evaluation^24^.

The use of different approaches in this study is due to the need for more consensus regarding the most appropriate methodologies for screening and identifying sarcopenia^46^. In 2016, Barbosa-Silva and colleagues proposed the SARC-F + CC (or SARC- CalF score), a modified version of the SARC-F, to improve its performance^34^. The SARC-CalF adds an anthropometric marker (CalF circumference) to the muscle functionality markers present in the original SARC-F^20,34,35,55,56^. CalF circumference assessment is a simple procedure of measuring the widest part of the right CalF with a non-elastic flexible plastic tape^24^. In older populations, the CalF circumference is measured as the most sensitive anthropometric index of muscle mass^57^.

The results of this investigation are similar to the findings demonstrated in previous studies, namely (a) a greater suspicion (screening) of sarcopenia in women than in men, and no differences in the objective identification of sarcopenia (assessment) were identified^16,21,58,59^; (b) a higher number of cases who suspected sarcopenia, with SARC-CalF than with SARC-F^11,24^; (c) a better diagnostic accuracy of sarcopenia of SARC-CalF compared to SARC-F having the EWGSOP2 as the gold standard^35,58,60^; (d) screening instruments showed higher specificity than sensitivity^11,35,56,58,60–62^. Age is a factor that favours the development of sarcopenia since the prevalence of symptoms is higher in older people^34,63,64^. In addition to age, sex also seems to be a determinant for the differences in the prevalence of sarcopenia observed in older adults: a lower muscle mass and lower use of muscle during the aging process (less physical activity) are likely explanations^65–67^.

This study has several limitations, namely the sample's representativeness (age group, sex, socio-economic status, residential area). For example, most participants were women, and sex plays an essential role in sarcopenia^68,69^. On the other hand, we limited our cross-sectional study to a comparison of SARC-F and SARC-CalF; a comparison between different sarcopenia screening approaches to predict important health outcomes such as disability, frailty, quality of life, and mortality should be investigated in prospective studies in the future. As strengths of this work, we highlight the investigation with a peculiar and little-studied sample whose participants live in poor cities with difficult access in Brazil, where screening is even more critical for health promotion and facilitation of clinical practice. However, this is the first study that compares the diagnosis of the two main sarcopenia screening instruments in the elderly population of Amazonas, including cutoff points for (not) suspected sarcopenia (SARC-Calf = 10.5 pts). Despite the promising results found in this study, its validity will need to be confirmed in further studies.

## Conclusion

Using walking slowness (≤ 8 m/s) as a reference method for identifying sarcopenia, the SARC-CalF performed better than the SARC-F for screening sarcopenia in the population ≥ 60 years of age in Amazonas, Brazil. Further studies are needed to verify this finding in other population groups and, above all, continue research to improve the performance of screening instruments.

## Data Availability

The data presented in this study are available upon request from the corresponding author. The data are not publicly available as they belong to a Ph.D. thesis in progress.
